# A Multipurpose and Multilayered Microneedle Sensor for Redox Potential Monitoring in Diverse Food Analysis

**DOI:** 10.3390/bios12111001

**Published:** 2022-11-10

**Authors:** Samuel M. Mugo, Dhanjai, Weihao Lu, Scott Robertson

**Affiliations:** 1 Department of Physical Sciences, MacEwan University, Edmonton, AB T5J 4S2, Canada; 2 Department of Chemistry, University of Allahabad, Prayagraj 211 002, India

**Keywords:** electrochemical microneedle sensor, MN redox sensor, food safety, H_2_O_2_, putrescine, food quality monitoring

## Abstract

This work presents a multipurpose and multilayered stainless steel microneedle sensor for the in situ redox potential monitoring in food and drink samples, termed MN redox sensor. The MN redox sensor was fabricated by layer-by-layer (LbL) approach. The in-tube multilayer coating comprised carbon nanotubes (CNTs)/cellulose nanocrystals (CNCs) as the first layer, polyaniline (PANI) as the second layer, and the ferrocyanide redox couple as the third layer. Using cyclic voltammetry (CV) as a transduction method, the MN redox sensor showed facile electron transfer for probing both electrical capacitance and redox potential, useful for both analyte specific and bulk quantification of redox species in various food and drink samples. The bulk redox species were quantified based on the anodic/cathodic redox peak shifts (E_a_/E_c_) on the voltammograms resulting from the presence of redox-active species. The MN redox sensor was applied to detect selected redox species including ascorbic acid, H_2_O_2_, and putrescine, with capacitive limits of detection (LOD) of 49.9, 17.8, and 263 ng/mL for each species, respectively. For the bulk determination of redox species, the MN redox sensor displayed LOD of 5.27 × 10^3^, 55.4, and 25.8 ng/mL in ascorbic acid, H_2_O_2_, and putrescine equivalents, respectively. The sensor exhibited reproducibility of ~1.8% relative standard deviation (%RSD). The MN redox sensor was successfully employed for the detection of fish spoilage and antioxidant quantification in king mushroom and brewed coffee samples, thereby justifying its potential for food quality and food safety applications. Lastly, the portability, reusability, rapid sampling time, and capability of in situ analysis of food and drink samples makes it amenable for real-time sensing applications.

## 1. Introduction

Reactive oxygen species (ROS) are natural by-products of mitochondrial aerobic respiration and cellular metabolism [1,2,3]. A balance of metabolic redox species comprising ROS and antioxidants maintain the cellular physiological metabolic functions in plants and animals at a steady state [4]. Due to both endogenous and exogenous causes, an imbalance of these redox species can lead to cellular oxidative stress [5,6]. To maintain physiological metabolic balance, the natural antioxidant defense system can inhibit the oxidation of ROS, thus preventing the release of free radicals [7,8,9]. Common antioxidants, such as ascorbic acid and thiols, participate in the termination of harmful chain oxidative processes caused by these free radicals [10].

Biogenic polyamines such as spermine, spermidine, and putrescine are by-products produced by the bacteria-catalyzed degradation of fruits, vegetables, and meats, and are an index of food freshness and quality [10,11]. Measuring the content of oxidants and antioxidants (reductants) in various foods can be a useful index for spoilage and fruit ripening [12], viral and bacterial infections onset, and as functional foods/nutraceuticals characterization [7]. In general, colorimetric assays utilizing 2,2-Diphenyl-1-picrylhydrazyl (DPPH) (LOD = 60.0–910 ng/mL with HPLC-DAD) [13] are commonly used for quantifying the antioxidant capacity of food and drink products [14]. Other techniques for the detection of oxidants and antioxidants in biological and environmental samples include nano-LC-ESI-MS (LOD = 6.00 × 10^3^ ng/mL), HPLC-UV-Vis (LOD = 30.0 ng/mL) [15], GC-MS/MS (LOD = 0.00814–0.0255 ng/mL) [16], and other spectroscopic techniques. However, all these methods are either tedious, require large amounts of reagents, or are susceptible to sample matrix interferences from chromophore species. Furthermore, these techniques are costly and not suitable for real time point-of-need detection applications [17]. Conversely, potentiometric probes for oxygen reduction potential (ORP) monitoring have been developed and commercialized. However, these probes are low in sensitivity and can provide only redox potential values without giving any insight on the causative redox species in the sample. Additionally, the instrumentation is bulky and not amenable for use in limited sample environments and non-invasive in situ monitoring.

**Figure 1:**
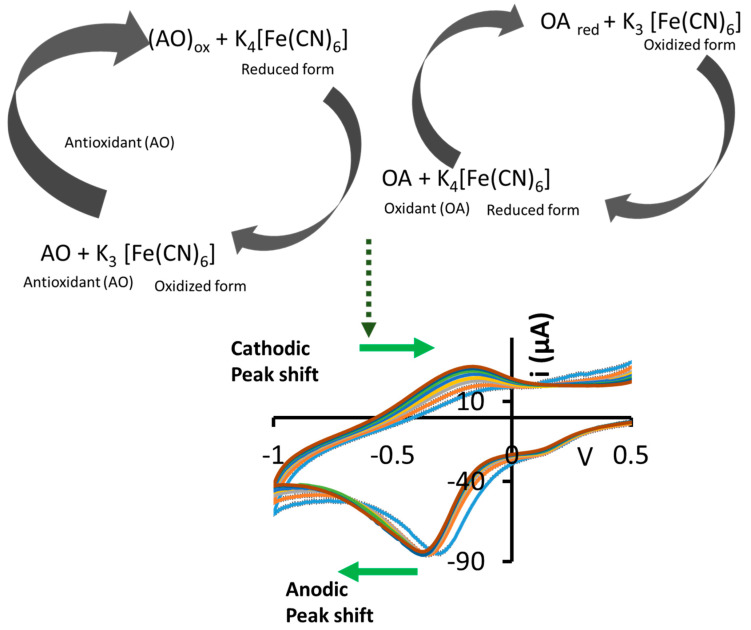
Illustration of the K_3_[Fe(CN)_6_]/K_4_[Fe(CN)_6_] redox probe reactions and resulting anodic/cathodic redox potential shifts in voltammograms.

Research has been conducted in the development of non-invasive platforms for redox potential monitoring due to their desirability. For instance, Brainina et al., 2016 [5], demonstrated a gel impregnated with the K_3_[Fe(CN)_6_]/K_4_[Fe(CN)_6_] redox couple (RC) that was taped on the skin for monitoring oxidant and antioxidant activity. In their study [5], CV was employed to record the anodic and cathodic response of K_3_[Fe(CN)_6_]/K_4_[Fe(CN)_6_] RC relative to the concentration of the oxidants and antioxidants. The reaction scheme of this process is illustrated in Figure 1. If ascorbic acid, a reducing agent and antioxidant, is present in the sample, it will induce the reduction of the redox couple from [Fe(CN)_6_]^3−^ to [Fe(CN)_6_]^4−^, and become oxidized to dehydroascorbic acid in the process [4,18]. On the other hand, if an oxidising agent such as H_2_O_2_ is present, [Fe(CN)_6_]^4−^ is oxidized to [Fe(CN)_6_]^3−^, with concomitant production of H_2_O and O_2_ [3,19].

This paper demonstrates a simple, robust, and reproducible sensing platform for rapid detection of redox potential, with the capacity for identification and quantitation of the oxidant and antioxidant content in food species. Designated as MN redox sensor, the sensor was fabricated by layer-by-layer (LbL) approach. Using stainless steel hypodermic needles as the substrate, the in-tube multilayer coatings comprised a CNT/CNC composite layer and a PANI layer intercalated with the K_3_[Fe(CN)_6_]/K_4_[Fe(CN)_6_] RC. Using a Bluetooth portable potentiostat, the MN redox sensor facilitated the electron transfer reactions for oxidation and reduction of the RC via CV. The redox potential sensing mechanism was based on the changes in the concentration ratio between the oxidized and reduced forms of the RC, which fluctuates depending on the presence of oxidant or antioxidant species. The fluxing concentration ratio resulted in the anodic (ΔE_a_) and cathodic (ΔE_c_) potential peak shifts of the RC in the resulting voltammograms. The variation in the capacitance and potential peak shift magnitude as a function of concentration were evaluated for common oxidizing and reducing agents, namely ascorbic acid, H_2_O_2_, and putrescine. Ascorbic acid is conventionally used as a surrogate standard for the quantification of antioxidants in foods, while putrescine is a common polyamine indicative of food spoilage, especially in meats [10,11,17]. The performance of the MN redox sensor was further evaluated for the quantification of redox potential in food samples including rotting fish, king mushroom, and brewed coffee.

## 2. Experimental Section

### 2.1. Materials and Reagents

Sodium phosphate, H_2_O_2_ (29%), putrescine, gallic acid, sulfuric acid (H_2_SO_4_, 98%), 30% H_2_O_2_, 3-(trimethoxysilyl) propyl methacrylate (TPM), methanol, aniline, ammonium persulfate (APS), ascorbic acid, monobasic dihydrogen phosphate (NaH_2_PO_4_), dibasic monohydrogen phosphate (Na_2_HPO_4_), potassium ferricyanide (K_3_[Fe(CN)_6_]), potassium chloride (KCl), glacial acetic acid, and (3-glycidyloxypropyl) trimethoxy silane (GOPS) were purchased from Sigma-Aldrich, Oakville, ON, Canada. The carboxylic acid functionalized multiwalled carbon nanotubes (CNTs) (optical diameter; 4–6 nm, 98%) were purchased from Times NanoChina. Cellulose nanocrystals (CNC) powder was donated by Alberta Innovates, Edmonton, Canada. Stainless steel hypodermic needles (0.7 × 40 mm, inner diameter 0.5 ± 0.1 mm) were bought from a local pharmacy. Salmon, king mushroom, and coffee samples were purchased from local market in Edmonton, Alberta. All aqueous solutions were prepared using de-ionized (DI) >18 MΩ Milli-Q water (Millipore, Bedford, MA, USA). All reagents were of analytical grade.

### 2.2. Fabrication of the MN Redox Sensor

First, the stainless-steel hypodermic needles were coated with a conductive layer by adapting our previous methodology with some modifications [20]. Briefly, the stainless-steel microneedles were immersed into piranha solution consisting of 1:1 H_2_SO_4_:H_2_O_2_ (*v*/*v*), which functionalized the metal surface with active hydroxyl moieties. The microneedles were then silylated by immersion in an aqueous solution consisting of TPM, DI water, and methanol in a 2:1:8 (*v*/*v*) ratio for 4 h. Silylation resulted in chemical binding of organosilane moieties to the microneedle surface. Then, 1.5 mL of a CNT/CNC (0.1%/0.4%) homogenous suspension (in DI water) was infused into the needle by a syringe pump at a flow rate of 15 µL/min leading to the formation of a thin composite layer of CNTs/CNCs within the microneedles. In the next step, the PANI conductive layer was polymerised within the CNT/CNC-coated needle at a low temperature. 1.5 mL of 0.1 M aniline prepolymer mixture (in 1 M H_2_SO_4_) was mixed with 50 µL of 0.1 M APS initiator and infused (at 15 µL/min) through the CNT/CNC needle incubated in an ice bath (4 °C) for polymerization to ensue. To integrate the RC into the in-tube PANI film, 1.5 mL of 25 mM K_3_[Fe(CN)_6_] (dissolved in 0.1 M KCl) was infused (15 µL/min) into the microneedle. Finally, to anchor the conductive layers to the salinized stainless-steel surface, a solution containing 0.5 mL of 20% GOPS solution (in 6 M glacial acetic acid) was infused through the microneedle and allowed to dry overnight at room temperature, followed by rinsing with deionized water. GOPS acted as a binding agent to stabilize the layers on the metal surface. Here, the fabricated RC-PANI@CNT/CNC microneedle was termed as MN redox sensor. Other sensors, such as the PANI@CNT/CNC MN and CNT/CNC microneedles were also fabricated using the same methodology for further study.

### 2.3. Instrumentation

CV and electrochemical impedance spectroscopy (EIS) were performed using BASi Palmsens-4 potentiostat (PalmSens B.V., Houten, The Netherlands). A three-electrode system was used for all electrochemical measurements, with the MN redox sensor, a platinum wire, and an in-house Ag/AgCl electrode [20] being used as working, auxiliary, and reference electrodes, respectively. The morphological characterizations of the sensors were carried out using Zeiss Sigma 300 VP field emission scanning electron micrograph (SEM) and energy dispersive X-ray spectroscopy (EDS) with LaB6 electron source (resolution ~100 nm). Moreover, HORIBA Raman spectrometer was used at 532 nm for evaluating structural properties of the sensors.

### 2.4. Electrochemical Characterization of the MN Redox Sensor

The electroactive surface area of the fabricated sensors was calculated by performing CV at different scan rate from 25 to 500 mV/s in 5 mM K_3_[Fe(CN)_6_], and the data were modelled with Randles–Sevcik equation. Furthermore, all the microneedle sensors were characterized using EIS to obtain the corresponding electron transfer resistances (R_ct_). EIS was performed in 5 mM K_3_[Fe(CN)_6_] in the 20.0–200,000.0 Hz frequency range, with a sinusoidal amplitude of 6.0 mV.

### 2.5. Voltammetric Quantification of Oxidant and Antioxidant Compounds Using the MN Redox Sensor

Ascorbic acid, gallic acid, H_2_O_2_, and putrescine were selected as standards to evaluate the performance of the PANI@CNT/CNC and MN redox sensor. Ascorbic acid, gallic acid, and H_2_O_2_ standards were prepared in a 9:1 solution of 0.1 M phosphate buffer (pH 7.0):1 M KCl, while putrescine standards were prepared in DI water. Each standard was spiked incrementally into 10 mL of a solution comprising 9 mL 0.1 M phosphate buffer:1 mL 1 M KCl, with four CVs (three for putrescine analysis) being acquired after 1 min of equilibration. The Δcapacitance and ΔE_c_ or ΔE_a_ were plotted linearly as a function of ascorbic acid, H_2_O_2_, or putrescine concentration.

All CVs were performed in the −1.0 to 1.0 V range, at a scan rate of 0.1 V/s. Faradaic capacitance was calculated by averaging the current within the −0.25 to −0.75 V (anodic), or the −0.5 to 0 V (cathodic) ranges and dividing it by the scan rate. The ∆capacitance was determined by subtracting the faradaic capacitance generated for the blank from the faradaic capacitance generated for the sample and dividing that value by the faradaic capacitance of the blank. Anodic (ΔE_a_) or cathodic (ΔE_c_) peak shifts were calculated by first determining the voltage at which the CV current peaks within either the anodic or cathodic voltage ranges, respectively. To determine peak shift values, the peak voltages were simply subtracted from the peak voltage obtained from the blank. ΔCapacitance and ΔE_a_/ΔE_c_ were used as the electrochemical signals for all experiments. Limits of detection (LOD) were determined by 3 times standard deviation of blank divided by sensor calibration sensitivity.

### 2.6. Antioxidant and ORP Quantification and Fish Spoilage Analysis Using the MN Redox Sensor

The MN redox sensor was evaluated for application in food quality monitoring by probing food or drink samples including coffee, king mushroom extract, and salmon. The coffee sample was analyzed without any additional sample preparation. The mushroom extract was prepared by blending 50 g of mushroom in 50 mL of ethanol and filtering out the solids. To get the fish in different stages of freshness, 5 g of fish tissue flesh was kept at room temperature for 1–5 days. Prior to analysis, each sample was first homogenized in 10 mL of DI water, sonicated for 30 min, and then filtered by cotton wool.

Samples were electrochemically analyzed by first employing the MN redox sensor to acquire the CV curves of blank electrolyte (9 mL 0.1 M phosphate buffer:1 mL 1 M KCl). The CV curves were then measured after spiking the blank electrolyte with 1 mL of sample solution, followed by the incremental spiking of ascorbic acid standard (in the case of king mushroom extract), gallic acid standard (in the case of coffee) and putrescine standard (in the case of fish extract). Four CV curves were acquired for all measurements after 1 min of equilibration time, except for fish extract analysis, where three CV curves were acquired.

## 3. Results and Discussion

### 3.1. Morphological and Electrochemical Characterization of MN Redox Sensor

**Figure 2:**
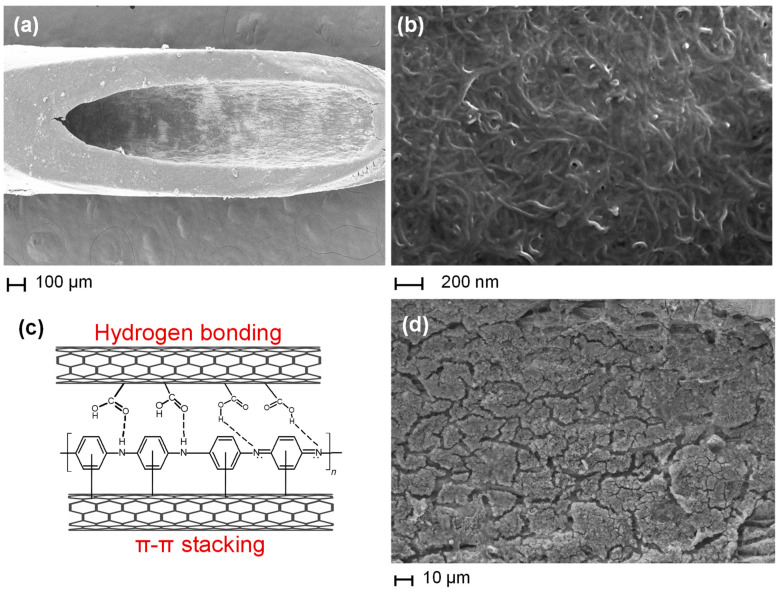
SEM images of the (**a**) CNT/CNC microneedle; and (**b**) enlarged view of open tips of the CNT/CNC microneedle and (**c**) Interaction of PANI and carboxylic functionalized CNT (**d**) SEM image of MN redox sensor.

The morphology of the MN redox sensor at different stages of LbL assembly was studied using SEM. The wall of the stainless-steel microneedle exhibits a typical fibrous, cross-linked network after addition of the CNT/CNC coating (Figure 2a,b) [20]. Following aniline polymerization, the PANI microparticles integrate within the porous structure of the CNT/CNC conductive layer. The resulting composite film is very stable due to the π-π interactions between the aromatic structures of PANI and the multiwalled CNTs [20]. The stability and conductivity of the PANI@CNT/CNC needle can also be attributed to hydrogen bonding between amides of PANI and carboxyl groups of CNTs as represented in Figure 2c [20]. Lastly, the thin K_3_[Fe(CN)_6_]/K_4_[Fe(CN)_6_] RC layer is shown to deposit onto the PANI nanoparticles of the PANI@CNT/CNC microneedle (Figure 2d). The iron within the K_3_[Fe(CN)_6_]/K_4_[Fe(CN)_6_] RC intercalates into the numerous amide and carboxyl groups of the PANI@CNT/CNC conductive layer via coordinate dative bonds.

**Figure 3:**
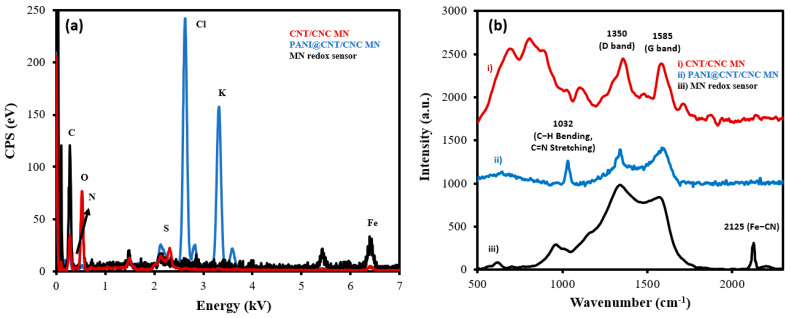
Overlapped (**a**) EDS and (**b**) Raman spectra taken from (i) CNT/CNC microneedle (MN); (ii) PANI@CNT/CNC MN; and (iii) MN redox sensor.

Successful LbL assembly of the MN redox sensor was confirmed by both EDS and Raman measurements taken at various stages of development. From the EDS spectra, the composition of the CNT/CNC layer was determined to be 38.8% of C and 61.2% of O (Figure 3a). The PANI@CNT/CNC microneedle contains 30.6% of C, 2.5% of N, 30.6% of O, and 6.5% of S (Figure 3a). The presence of N and S indicates the successful polymerization and incorporation of PANI onto the CNT/CNC matrix. Lastly, the MN redox sensor comprised of 9.6% of C, 3.4% of N, 4.0% of O, 38.4% of Cl, 41.2% of K, and 3.4% of Fe (Figure 3a). The increased percentage of N relative to the PANI@CNT/CNC microneedle, and the presence of 3.4% of Fe confirmed the integration of the K_3_[Fe(CN)_6_]/K_4_[Fe(CN)_6_] RC, whereas the high percentage of K and Cl came from the KCl of the RC solution.

Figure 3b shows the overlapped Raman spectra for the MN redox sensor during its stages of development. The CNT/CNC microneedle shows two distinct peaks at 1350 cm^−1^ and 1585 cm^−1^ (Figure 3b), which correspond to the D and G band of the CNTs, respectively [20,21,22,23]. The D band is attributed to disordered carbon structures, while the G band is due to the vibration of C-C bond stretching [20,21,22,23]. The PANI@CNT/CNC microneedle shows a peak at 1032 cm^−1^ (Figure 3b), ascribed to the C-H bending in benzenoid structure, and the C=N stretching in the quinoid structure of the PANI [24]. The MN redox sensor spectrum exhibits a sharp peak at 2125 cm^−1^ (Figure 3b), representing the ferricyanide group, confirming the successful integration of the K_3_[Fe(CN)_6_]/K_4_[Fe(CN)_6_] RC onto the PANI@CNT/CNC film [25].

**Figure 4:**
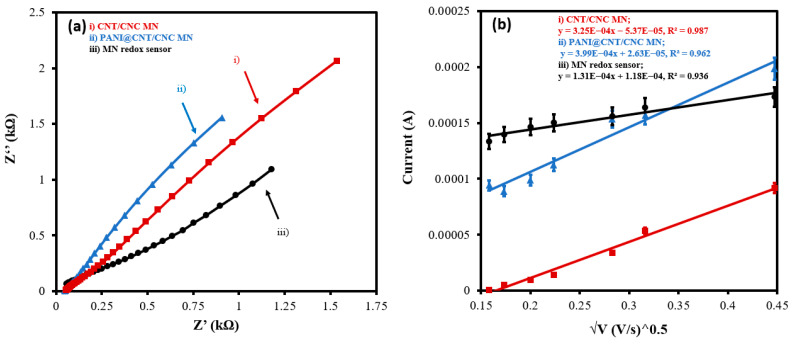
(**a**) Overlapped EIS Nyquist plots and (**b**) overlapped linear calibration plot of peak cathodic current vs. square root of scan rate for (i) CNT/CNC microneedle (MN); (ii) PANI@CNT/CNC MN; and (iii) MN redox sensor.

The MN redox sensor was further characterized by EIS using 5 mM potassium ferricyanide (in 0.1 M KCl) as the standard redox probe. Figure 4a shows the overlapped Nyquist plots of the MN redox sensor, PANI@CNT/CNC microneedle, and CNT/CNC microneedle. The R_ct_ of the different fabricated sensors was determined by fitting each Nyquist plot to an equivalent electrical circuit (Figure S1, Supplementary Materials). Lower values of R_ct_ are indicative of higher conductivity and more effective electric signal transduction. The R_ct_ values for CNT/CNC microneedle, PANI@CNT/CNC microneedle, and the MN redox sensor were found to be 347.6, 45.28, and 51.51 Ω, respectively (Figure S1). Addition of the PANI to the CNT/CNC microneedle significantly reduced the electron transfer resistance, enhancing the sensor conductivity. However, there is a slight decrease in R_ct_ value after potassium ferricyanide integration due to its higher electrical resistivity. The electroactive surface area for each stage of the MN redox sensor development was evaluated by performing CV at different scan rates from 25–500 mV/s and plotting the cathodic peak current as a function of the square root of the scan rate (Figure 4b). Data from the linear plots were fitted into the Randles–Sevcik equation to calculate the electroactive surface area of the microneedles [20,26,27]. The electroactive surface area for the CNT/CNC microneedle, PANI@CNT/CNC microneedle, and the MN redox sensor was determined to be 0.0070 ± 0.0004, 0.0210 ± 0.0005, and 0.0180 ± 0.0005 cm^2^, respectively.

### 3.2. Electrochemical Analysis of Oxidant, Antioxidant, and Polyamine Compounds Using MN Redox Sensor

**Figure 5:**
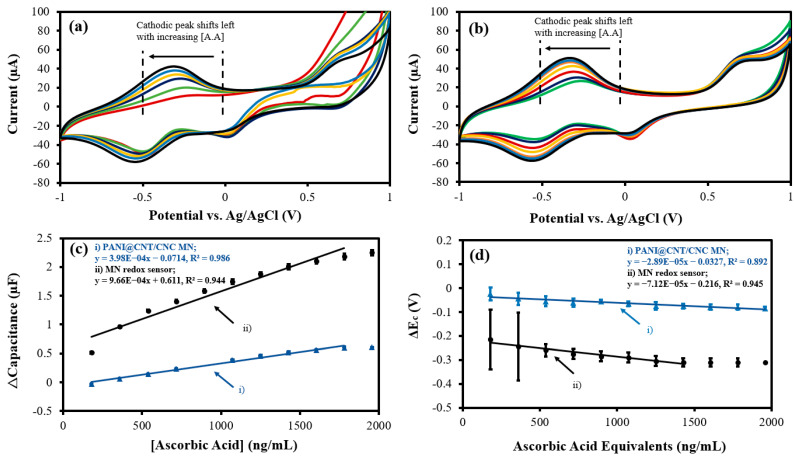
Representative overlapped CVs showing (**a**) MN redox sensor; and (**b**) PANI@CNT/CNC microneedle (MN) electrochemical response to 180–1.96 × 10^3^ ng/mL ascorbic acid (A.A) and resulting (**c**) Δcapacitance; and (**d**) ΔE_c_ vs. ascorbic acid concentration plots obtained using the (**i**) PANI@CNT/CNC microneedle (MN) and (**ii**) MN redox sensor.

The MN redox sensor was used to electrochemically analyze and quantify an antioxidant (ascorbic acid), an oxidant (H_2_O_2_), and a diamine (putrescine). The overlapped CV responses of the MN redox sensor and PANI@CNT/CNC MN to 180–1.96 × 10^3^ ng/mL of ascorbic acid are shown in Figure 5a,b, respectively. Notably, the cathodic peak generated from both sensors increases in magnitude and shifts left with increasing concentrations of ascorbic acid (Figure 5a,b), indicating the redox of the antioxidant. Accordingly, the cathodic Δcapacitance and ΔE_c_ vs. ascorbic acid concentration plots for both sensors are shown in Figure 5c,d. For the capacitive quantification of ascorbic acid, the MN redox sensor had a calibration sensitivity approximately 143% greater than that of the PANI@CNT/CNC MN (Table 1). Additionally, the LOD for ascorbic acid for capacitive detection was 121 ng/mL and 49.9 ng/mL for PANI@CNT/CNC MN and the MN redox sensor, respectively. Conversely, the ΔE_c_ metric represents the ORP of the solution, and may be used for quantification of the total sum of redox species present. In this regard, the MN redox sensor displays a sensitivity approximately 146% larger than the PANI@CNT/CNC MN (Table 1). The LOD for bulk redox species detection was 2.75 × 10^3^ and 5.27 × 10^3^ ng/mL ascorbic acid equivalents for the MN redox sensor and PANI@CNT/CNC MN, respectively. The detection ranges for the MN redox sensor are 180–1.78 × 10^3^ ng/mL ascorbic acid, and 180–1.43 × 10^3^ ng/mL ascorbic acid equivalents for capacitive and ORP detection, respectively.

**Figure 6:**
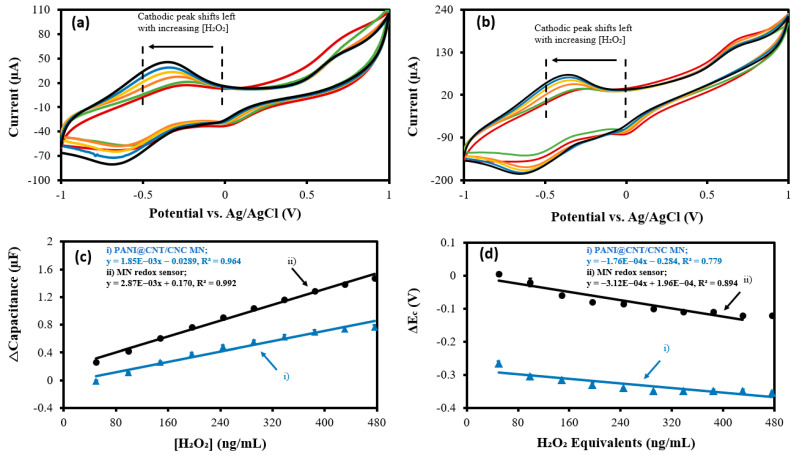
Representative overlapped CVs showing (**a**) MN redox sensor; and (**b**) PANI@CNT/CNC microneedle (MN) electrochemical response to 49.8–476 ng/mL H_2_O_2_ and resulting (**c**) cathodic Δcapacitance; and (**d**) ΔE_c_ vs. H_2_O_2_ concentration plots obtained using the (**i**) PANI@CNT/CNC microneedle (MN) and (**ii**) MN redox sensor.

CVs obtained using both the MN redox sensor and PANI@CNT/CNC MN show a significant cathodic redox peak, which shift leftwards with increasing concentrations of H_2_O_2_ (Figure 6a,b). The resulting cathodic Δcapacitance and ΔE_c_ vs. H_2_O_2_ concentration plots are shown in Figure 6c,d, respectively. The sensitivity of the MN redox sensor was approximately 55.1% higher compared to that of the PANI@CNT/CNC MN (Table 1), indicating improved performance for capacitive quantification of H_2_O_2_. For detection of ORP (in H_2_O_2_ equivalents), the MN redox sensor once again shows enhanced performance, being 77.3% more sensitive than the PANI@CNT/CNC MN (Table 1). For capacitive detection of H_2_O_2_, the LOD were found to be 14.3 and 17.8 ng/mL for the MN redox sensor and PANI@CNT/CNC MN respectively. For bulk redox species detection, the LOD were 55.4 and 98.4 ng/mL H_2_O_2_ equivalents for the MN redox sensor and PANI@CNT/CNC MN, respectively. Lastly, the detection ranges for the MN redox sensor are 49.8–476 ng/mL H_2_O_2_, and 49.8–431 ng/mL H_2_O_2_ equivalents for capacitive and ORP detection, respectively. These results indicate the utility of both sensors for use in oxidant and antioxidant quantification.

**Figure 7:**
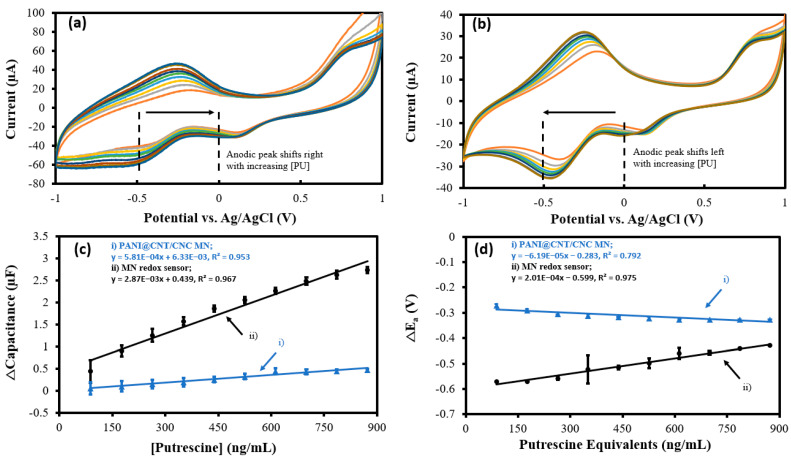
Representative CVs showing (**a**) MN redox sensor; and (**b**) PANI@CNT/CNC microneedle (MN) electrochemical response to 88.0–873 ng/mL putrescine and resulting (**c**) cathodic Δcapacitance; and (**d**) ΔE_a_ vs. putrescine concentration calibration plots obtained using the (**i**) PANI@CNT/CNC microneedle (MN) and (**ii**) MN redox sensor.

Putrescine is a common indicator of microbial food spoilage and is an antioxidant compound with reductive properties [28,29]. Overlapped CVs obtained using the MN redox sensor and PANI@CNT/CNC microneedle in the 88.0–873 ng/mL putrescine range are shown in Figure 7a,b, respectively. A shift in the anodic redox peak potential was observed (Figure 7a,b), indicating the oxidation of putrescine on the MN redox sensor and PANI@CNT/CNC microneedle surface [30,31]. The resulting cathodic Δcapacitance and ΔE_a_ vs. putrescine concentration calibrations are shown in Figure 7c,d, respectively. The MN redox sensor demonstrated a 393% increase in sensitivity for capacitive quantification of putrescine compared to the PANI@CNT/CNC microneedle (Table 1). The LOD for the capacitive quantification of putrescine using CV was determined to be 711 and 263 ng/mL for the PANI@CNT/CNC MN and MN redox sensor, respectively. For bulk redox species detection, the MN redox sensor displayed enhanced performance, having a sensitivity 224% greater compared to the PANI@CNT/CNC MN (Table 1). Additionally, the LOD for the bulk redox species detection was found to be 280 and 25.8 ng/mL putrescine equivalents for the PANI@CNT/CNC MN and MN redox sensor, respectively. The detection range for the MN redox sensor is 88.0–873 ng/mL putrescine, and 88.0–873 ng/mL putrescine equivalents for capacitive and ORP detection, respectively. The capacity of the MN redox sensor to respond to putrescine demonstrates its applicability for fish spoilage monitoring.

Table 2 outlines the various concentration ranges and LODs of various electrochemical sensors for the analytes under analysis. Despite the linear detection range of the MN redox sensor being narrower, the lower limit of the detection range is less than several reported electrochemical sensors (Table 2), making low-concentration measurements more accurate. Moreover, the LODs of each analyte are either lower, or comparable to some of the reported sensors (Table 2). While conventional chromatographic techniques, may yield highly accurate results, low LODs, and wider detection ranges (Table 2), their operation is tedious and non-amenable to rapid antioxidant determination [17,32]. Conversely, the MN redox sensor is portable, reusable, takes rapid measurements, and is capable of in situ analysis of food and drink samples. While the MN redox sensor has several advantages, it is limited in availability due to the requirement of a needle infuser pump to fabricate.

**Table 1:** Capacitive and ORP sensitivities for the PANI@CNT/CNC microneedle (MN) and MN redox sensor in response to ascorbic acid, H_2_O_2_, and putrescine analytes.

Analyte	PANI@CNT/CNC MN Capacitive Sensitivity (µF·mL/ng)	MN Redox Sensor Capacitive Sensitivity (µF·mL/ng)	PANI@CNT/CNC MN ORP Sensitivity (V·mL/ng)	MN Redox Sensor ORP Sensitivity (V·mL/ng)
Ascorbic Acid	3.98 × 10^−4^	9.66 × 10^−4^	−2.89 × 10^−5^	−7.12 × 10^−5^
H_2_O_2_	1.85 × 10^−3^	2.87 × 10^−3^	−1.76 × 10^−4^	−3.12 × 10^−4^
Putrescine	5.81 × 10^−4^	2.87 × 10^−3^	−6.19 × 10^−5^	2.01 × 10^−4^

**Table 2:** Literature comparison of performance metrics of electrochemical and conventional redox analyte determination methods to the reported MN redox sensor.

Sensing Method	Analyte	Concentration Range (ng/mL)	LOD (ng/mL)	Reference
3-D nitrogen-doped graphene-modified GCE	Ascorbic Acid	3.52 × 10^3^–1.76 × 10^6^	689	[33]
K_4_Fe(CN)_6_-doped Ppy-modified platinum electrode	Ascorbic Acid	176–1.76 × 10^4^	44.0	[34]
K_3_[Fe(CN)_6_]/K_4_[Fe(CN)_6_]-infused PANI@CNT/CNC MN electrode	Ascorbic Acid	180–1.78 × 10^3^ (Capacitive) 180–1.43 × 10^3^ ascorbic acid equivalents (ORP)	49.9 (Capacitive) 2. 75 × 10^3^ ascorbic acid equivalents (ORP)	This work
Screen-printed carbon electrode modified with a carboxylated triazole copper complex	H_2_O_2_	340–1.78 × 10^4^	19.4	[35]
Copper oxide/graphitic carbon nitride-modified GCE	H_2_O_2_	17.0–1.70 × 10^3^	10.5	[36]
K_3_[Fe(CN)_6_]/K_4_[Fe(CN)_6_]-infused PANI@CNT/CNC MN electrode	H_2_O_2_	49.8–476 (Capacitive) 49.8–431 H_2_O_2_ equivalents (ORP)	14.3 (Capacitive) 55.4 H_2_O_2_ equivalents (ORP)	This work
K_4_Fe(CN)_6_-doped Ppy-modified screen-printed carbon electrode	Putrescine	88.2–8.82 × 10^3^	30.0	[37]
Diamine oxidase, Prussian blue, and indium tin oxide nanoparticle-modified screen-printed carbon electrode	Putrescine	696–2.64 × 10^5^	688	[38]
K_3_[Fe(CN)_6_]/K_4_[Fe(CN)_6_]-infused PANI@CNT/CNC MN electrode	Putrescine	88.0–872 (Capacitive) 88.0–872 putrescine equivalents (ORP)	263 (Capacitive) 25.8 putrescine equivalents (ORP)	This work
Nano-LC-ESI-MS and HPLC-UV-Vis	Anthocyanins	6.00 × 10^3^–5.00 × 10^4^ (Nano-LC-ESI-MS) 100–5.00 × 10^4^ (HPLC-UV-Vis)	6.00 × 10^3^ (Nano-LC-ESI-MS) 30.0 (HPLC-UV-Vis)	[15]
HPLC-DAD	Tea antioxidants	Several	60.0–910	[13]
Modified GC-MS/MS method	Phenolic antioxidants	0.100–1.00	0.00814–0.0255	[16]

### 3.3. Determination of Antioxidant Content in King Mushroom and Brewed Coffee Samples

**Figure 8:**
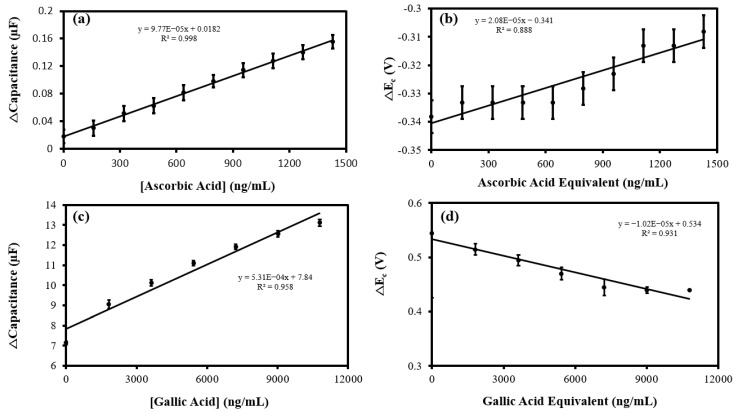
(**a**) Cathodic Δcapacitance; and (**b**) ΔE_c_ vs. ascorbic acid concentration standard addition (SA) calibration plots of king mushroom sample analysis (*n* = 3) using the MN redox sensor. (**c**) Cathodic Δcapacitance; and (**d**) ΔE_c_ vs. gallic acid concentration SA calibration plots of brewed coffee sample analysis (*n* = 3) using the MN redox sensor.

The MN redox sensor was used to detect and quantify ascorbic acid and gallic acid in king mushroom extract and brewed coffee, respectively. King mushrooms contain high levels of antioxidants in the form of the ergothioneine amino acid [39]. Nutrients contained within king mushrooms have strong ROS scavenging abilities, and effectively chelate ferrous ions [40,41]. Typically, king mushrooms contain 5–15 mg/100g of ascorbic acid, which varies due to the trophic component of soil [42]. To determine the ascorbic acid concentration and the total sum of redox-active species within the king mushroom sample, capacitive (Figure 8a) and ΔE_c_ (Figure 8b) standard addition (SA) plots were generated from the CVs in Figure S2a. Ascorbic acid content within the mushroom was determined to be 2.04 × 10^3^ ng/mL using the Δcapacitance vs. ascorbic acid plot (Figure 8a). Using the ΔE_c_ vs. ascorbic acid equivalent plot (Figure 8b), the total sum of redox-active species within the mushroom extract was found to be 1.80 × 10^5^ ng/mL ascorbic acid equivalents. Coffee is a rich source of bioactive antioxidants, especially phenolic acids, which are highly beneficial to the consumer’s health [43]. The SA method was again employed to determine the gallic acid content and the total content of redox species in freshly brewed coffee. Representative voltammograms for the SA analysis are shown in Figure S2b. Additionally, the MN redox sensor has been demonstrated to respond linearly to gallic acid (Figure S3), confirming its ability to respond to the antioxidant. Using capacitive detection (Figure 8c), the gallic acid concentration in coffee was found to be 0.162 mg/mL, while ΔE_c_ detection method (Figure 8d) determined the sum of all oxidants and antioxidants to be 0.574 mg/mL gallic acid equivalents.

### 3.4. Electrochemical Monitoring of Fish Spoilage

**Figure 9:**
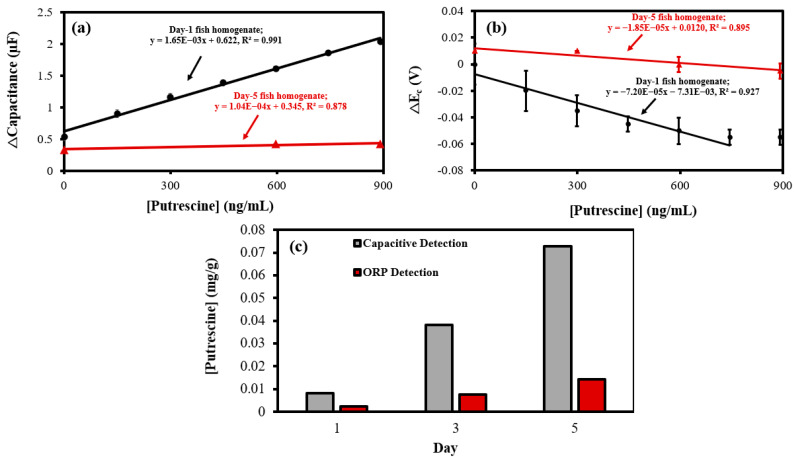
(**a**) Cathodic Δcapacitance; and (**b**) ΔE_c_ vs. putrescine concentration standard addition (SA) calibration plots of 1-day and 5-day-old salmon fish analysis (*n* = 3) using the MN redox sensor. (**c**) Putrescine concentrations calculated from cathodic Δcapacitance (Capacitive Detection) and ΔE_c_ (ORP Detection) vs. putrescine concentration SA plots in salmon taken over a 5-day period.

Putrescine is a biogenic amine responsible for the foul odor of putrefying flesh and can be used as an indicator for the microbial decay in meats [44]. The SA method was used to analyze putrescine concentrations in salmon homogenate sample every day over a five-day period. Representative CVs outlining the MN redox sensor response to blank phosphate buffer, day 1 and day 5 fish, and 892 ng/mL of putrescine are shown in Figure S4. The resulting cathodic Δcapacitance, and ΔE_c_ vs. putrescine concentration SA calibration curves for day 1 and day 5 fish samples are shown in Figure 9a,b, respectively. The day 1, 3, and 5 concentrations of putrescine in the salmon samples are shown in Figure 9c. On day 1, the putrescine concentration within the fish is relatively low, indicating little spoilage. As spoilage progresses, putrescine concentrations within the fish gradually increase for both capacitive and ORP detection, before peaking on day 5 (Figure 9c), indicating severe spoilage. This timeline aligns with the timeframe from Trevino’s experiment examining raw meat decay [45]. These inferences justify the utilization of MN redox sensor as food spoilage sensor.

## 4. Conclusions

The development of an alternative method to quantify the oxidant and antioxidant content in foodstuffs is important for food quality and food safety. A robust, multipurpose, and multilayered stainless steel microneedle sensor was developed using a PANI@CNT/CNC microneedle integrated with the K_3_[Fe(CN)_6_]/K_4_[Fe(CN)_6_] redox mediator system. The sensor was fabricated by the LbL method and was characterized using various techniques. EDS and Raman spectra of each layer confirmed the successful LbL fabrication of the sensor. The developed MN redox sensor exhibited high sensitivity to quantify the oxidant and antioxidant activity through a facile electron transfer mechanism for probing the redox potential. The fabricated sensor successfully detected and quantified ascorbic and gallic acid antioxidants in king mushroom and brewed coffee samples, respectively. Moreover, the MN redox sensor investigated fish spoilage by monitoring putrescine concentrations in salmon samples taken every day over a 5-day period. Overall, the sensor holds promise to act as a device for monitoring food quality and food safety. Future studies will include further tests on other biological samples, as well as the determination of multiple antioxidants in given samples.

## Data Availability

All relevant data is included in the manuscript and in the supporting information.

## Supplementary Materials

The following supporting information can be downloaded at: https://www.mdpi.com/article/10.3390/bios12111001/s1, Figure S1: EIS equivalent circuit fittings for the MN redox sensor at different stages of development; Figure S2: Representative overlapped CVs showing the MN redox sensor electrochemical response to (a) PBS blank, king mushroom extract, and 1.43 × 10^3^ ng/mL ascorbic acid (A.A); and (b) PBS blank, fresh brewed coffee, and 1.08 × 10^4^ ng/mL gallic acid (G.A) during standard addition analysis; Figure S3: (a) Representative overlapped CVs showing the MN redox sensor electrochemical response to 999–1.09 × 10^4^ ng/mL gallic acid, and resulting (b) cathodic Δcapacitance; and (c) ΔEc vs. gallic acid concentration plots obtained using the MN redox sensor; Figure S4; Representative overlapped CVs showing the MN redox sensor electrochemical response to PBS blank, day-1 fish homogenate, day-5 fish homogenate, and 892 ng/mL of putrescine during standard addition analysis.
