# Supplementary material for: A Multipurpose and Multilayered Microneedle Sensor for Redox Potential Monitoring in Diverse Food Analysis
Source: Biosensors (Basel). 2022 Nov 10;12(11):1001. doi: 10.3390/bios12111001 (PMC9688395; doi:10.3390/bios12111001)
Supplement: Supplementary file 1 [file biosensors-12-01001-s001.zip › biosensors-1967757-supplementary.pdf]

A Multipurpose and Multilayered Microneedle Sensor for Redox Potential Monitoring in Diverse Food Analysis

Samuel M. Mugo [1*], Dhanjai [2*], Weihao Lu [1] and Scott Robertson [1]

[1] *Department of Physical Sciences, MacEwan University, Edmonton, AB T5J4S2, Canada*

**Correspondence: mugos@macewan.ca; dhanjai83@gmail.com*

[2] *Department of Chemistry, University of Allahabad, Prayagraj 211002, India*

Figure S1. EIS equivalent circuit fittings for the MN redox sensor at different stages of development.

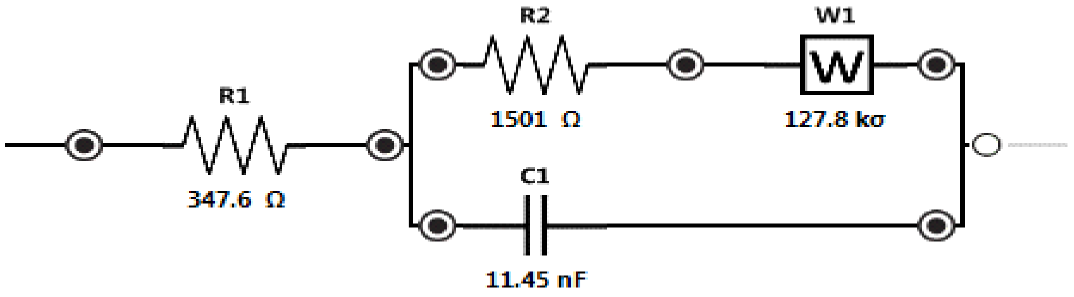

(a) CNT/CNC Microneedle

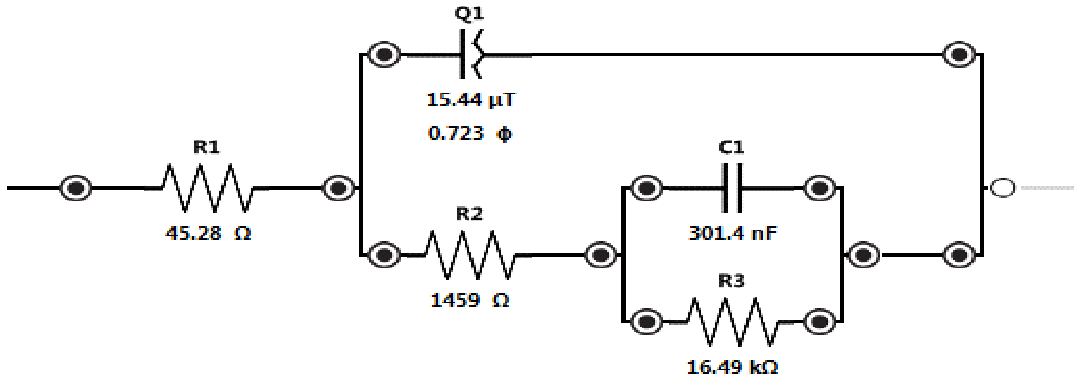

(b) PANI@CNT/CNC Microneedle

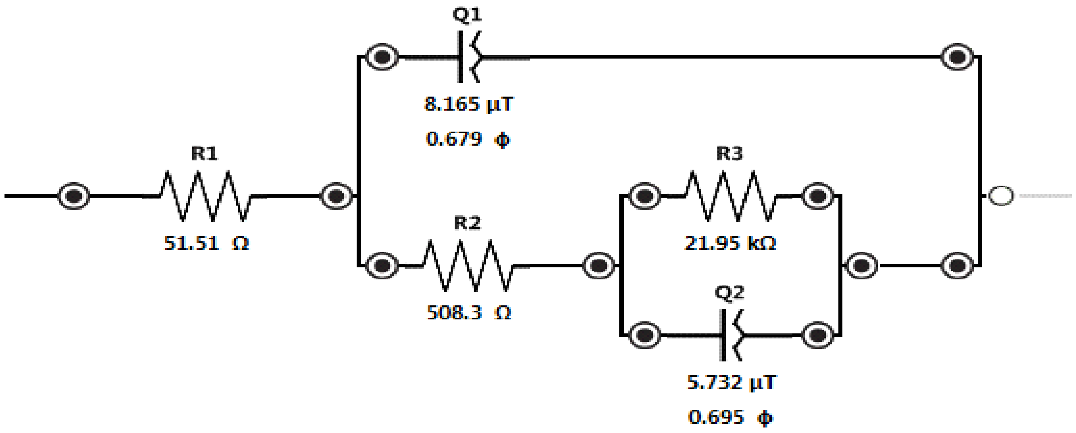

(c) MN redox sensor

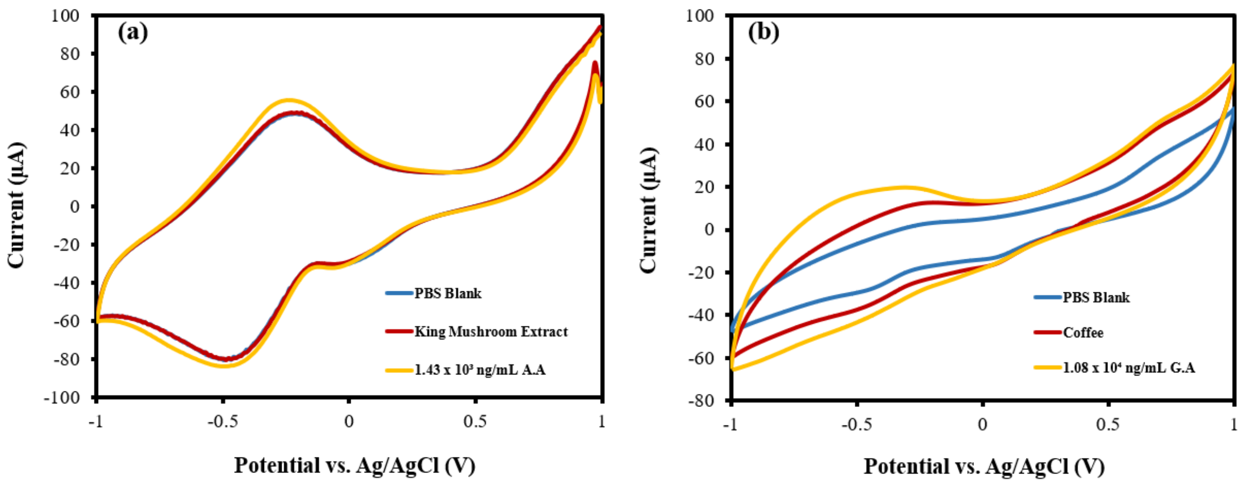

Figure S2. Representative overlapped CVs showing the MN redox sensor electrochemical response to **(a)** PBS blank, king mushroom extract, and 1.43 x 10^3 ng/mL ascorbic acid (A.A); and **(b)** PBS blank, fresh brewed coffee, and 1.08 x 10^4 ng/mL gallic acid (G.A) during standard addition analysis.

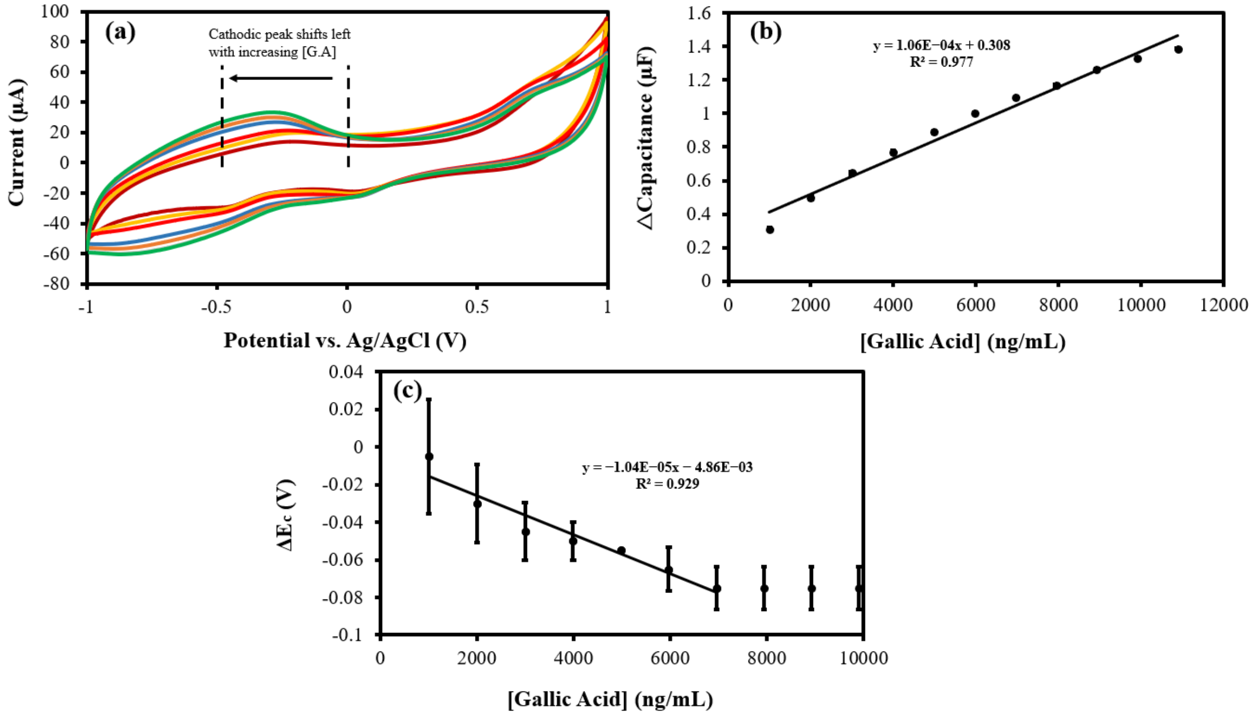

Figure S3. **(a)** Representative overlapped CVs showing the MN redox sensor electrochemical response to 999 – 1.09 x 10^4 ng/mL gallic acid, and resulting **(b)** cathodic Δcapacitance; and **(c)** ΔE_c vs. gallic acid concentration plots obtained using the MN redox sensor.

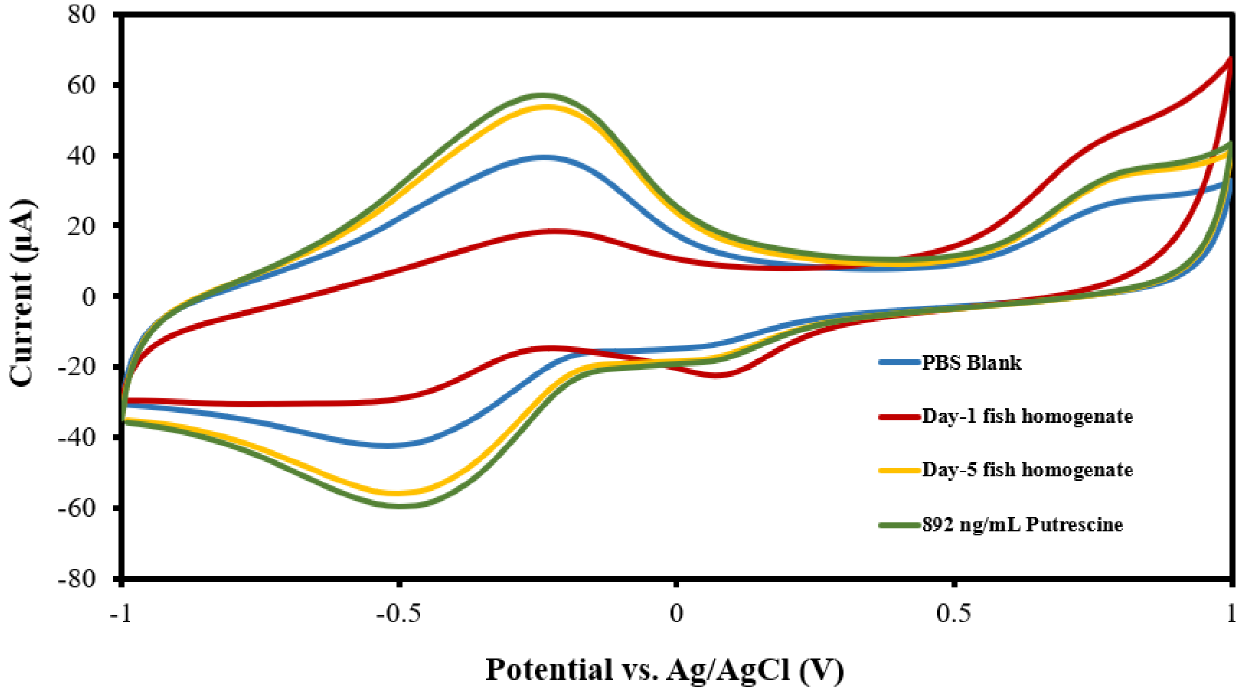

Figure S4. Representative overlapped CVs showing the MN redox sensor electrochemical response to PBS blank, day-1 fish homogenate, day-5 fish homogenate, and 892 ng/mL of putrescine during standard addition analysis.
